# Characterization of histone acetyltransferase and histone deacetylase genes under abiotic and hormone stresses in soybean

**DOI:** 10.3389/fpls.2026.1753615

**Published:** 2026-03-03

**Authors:** Kai Liu, Shang Sun, Enhui Guo, Xue Liu, Manman Duan, Hong Zhang, Chao Xue, Zhenlin Wei, Zhiyun Gong

**Affiliations:** 1 Shandong Key Laboratory of Biophysics, Institute of Rural Revitalization, Dezhou University, Dezhou, China; 2 Belgorod Institute of Food Sciences, Dezhou University, Dezhou, China; 3 School of Agronomy and Horticulture, Jiangsu Vocational College of Agriculture and Forestry, Zhenjiang, China; 4 Jiangsu Key Laboratory of Crop Genomics and Molecular Breeding/Zhongshan Biological Breeding Laboratory/Key Laboratory of Plant Functional Genomics of the Ministry of Education, Agricultural College of Yangzhou University, Yangzhou, China

**Keywords:** histone acetyltransferase, histone deacetylase, hormone treatment, soybean, stress

## Abstract

**Introduction:**

Histone acetyltransferases (HATs) and histone deacetylases (HDACs) dynamically regulate histone acetylation and are involved in the process of plant growth and development and stress responses.

**Methods:**

In this study, we identified 12 *GmHATs* and 28 *GmHDACs* in the soybean genome and systematically analyzed their phylogenetic relationships, structural features, expression profiles, and stress-induced acetylation dynamics using bioinformatics analysis, RT-qPCR, and western blotting.

**Results:**

*Cis-element* analysis indicates that they may participate in hormone and stress signaling pathways, and transcriptome analysis revealed tissue-specific expression patterns. RT-qPCR results indicated that *GmHATs* and *GmHDACs* exhibited varying degrees of induced expression under salt and drought stress, particularly the *GmHDA16* and *GmHDT2*. Notably, under salt stress, *GmHDT2* expression increased 61-fold. Western blotting further demonstrated that salt and drought treatments significantly reduced H3K18ac and H4K8ac levels. Additionally, these genes exhibit distinct responses to various plant hormones.

**Discussion:**

The reduction in acetylation was negatively correlated with the upregulation of HD2 subfamily genes, suggesting that specific HDACs mediate stress responses through histone deacetylation. This study provides new insights into the epigenetic regulation of abiotic stress in soybean, offering valuable genetic resources for future stress-resistant breeding programs.

## Introduction

As sessile growing organisms, plants are unable to escape environmental stresses by moving, and thus have evolved complex epigenetic regulatory mechanisms to respond rapidly to external changes. Histone acetylation, a highly conserved epigenetic modification, plays a central role in plant growth and development and environmental adaptation by dynamically regulating chromatin structure and gene transcriptional activity (Bertrand et al., 2003; Liang et al., 2025; Wu et al., 2023). This process is dynamically regulated by HATs and HDACs. HATs catalyze the acetylation of histone N-terminal lysine residues, which neutralizes the positive charge and attenuates their electrostatic binding to DNA, contributing to the relaxation of chromatin structure to activate transcription, whereas HDACs restore chromatin turgor by removing acetyl groups to achieve transcriptional repression.

Plant HATs can be divided into four major subfamilies based on their structure and subcellular localization: the GCN5-related N-terminal acetyltransferase (GNAT), CREB-binding protein (CBP), MOZ, Ybf2/Sas3, Sas2 and Tip60 superfamily (MYST), and TATA-binding protein-associated factor (TAFII250) (Pandey et al., 2002). HDACs are divided into three subclasses: the reduced potassium dependency 3/histone deacetylase 1 (RPD3/HDA1), histone deacetylase 2 (HD2), and silent information regulator 2 (SIR2) (Hollender and Liu, 2008). These enzymes regulate chromatin relaxation and gene transcriptional activation by catalyzing the acetylation of N-terminal lysine residues such as histone H3 and H4, and are involved in processes such as signal transduction, hypocotyl elongation, petal development, and stress response (Fang et al., 2025; Guo et al., 2020; Huang and Irish, 2024; Lee and Seo, 2019; Liu et al., 2023; Xu et al., 2018).

Salt and drought stress is a major threat to global agricultural production and ecosystem stability, leading to reduced crop yields, land degradation and loss of biodiversity, increasing food security and environmental sustainability challenges (Hussain et al., 2020). Several studies have found that epigenetic regulation mediated by HATs and HDACs plays a key role in plant response to salt and drought stress by dynamically modifying chromatin structure to regulate the expression of stress-related genes (Cheng et al., 2018; Li et al., 2019b; Zheng et al., 2019). In *Arabidopsis thaliana*, GCN5 regulates the expression of cellulose synthesis genes by altering the level of acetylation at H3K9 and H3K14 to maintain cell wall integrity and thus improve salt stress tolerance (Zheng et al., 2019). ZmGCN5 in *Zea mays* improves salt tolerance by increasing the levels of H3K9ac at the *ZmEXPB2* and *ZmXET1* promoters, causing root swelling (Li et al., 2014). In contrast, *OsHDA710* in rice (*Oryza sativa*) functions as a negative regulator of drought tolerance by directly binding to the TGACC motif within the promoters of drought-tolerance genes (Chen et al., 2025). The ABA-Responsive Element Binding Protein 1 (AREB1) and ADA2b-GCN5 complexes in *Populus trichocarpa* regulate the expression of drought-responsive genes (*PtrNAC006*, *PtrNAC007*, and *PtrNAC120*) by enhancing H3K9ac under drought stress conditions (Li et al., 2019b). Previous studies have indicated that multiple HDACs participate in plant responses to abiotic stress. Overexpression of the *HDA15* gene confers resistance to salt stress by regulating the levels of H3K14ac and H4K16ac in *NCED3* (Truong et al., 2021; Ueda et al., 2017). OsHDA706 is a histone H4 deacetylase in *Oryza sativa* that enhances salt tolerance by regulating the expression of *OsPP2C49* through H4K5 and H4K8 deacetylation (Liu et al., 2023). OsHDA704 binds directly to *DST* and *ABIL2* and represses their expression to positively regulate drought and salt tolerance (Zhao et al., 2021). Studies have suggested that HATs and HDACs are involved in plant signaling pathways. HDA15 negatively regulates the *RHO GTPASE* (*ROP*) gene in plants in ABA signaling by mediating the deacetylation of histones H3 and H4 and interacting with MYB96 (Lee and Seo, 2019). Similarly, HDA9 interacts with the PWR-ABI4 complex and acts on the *CYP707A1/2* genes encoding key enzymes in ABA metabolism through deacetylation, thereby promoting drought resistance development (Ali and Yun, 2020). Furthermore, HDT4 collaborates with the ENAP1-ENAP2-MYB44 complex to regulate drought-sensitive genes by modifying H3K27ac (Zhao et al., 2022). The GCN5-TPL-HDA6 module maintains homeostasis of acetylated *TPL* to regulate JA signaling (An et al., 2022). In the SA signaling pathway, the HAC-NPR1-TGA complex activates SA-dependent plant immunity by promoting *PR* transcription through histone acetylation (Han et al., 2007).

Soybean (*Glycine max*) an important grain, oil and feed crop, has yet to be comprehensively explored for its HATs and HDACs gene families. Here, we identified *G.max* HATs (GmHATs) and HDACs (GmHDACs) family member genes at the genomic level and analyzed their physicochemical properties, gene structures, conserved motifs, chromosomal distributions, covariance analysis, and *cis-elements*. Meanwhile, we conducted a comprehensive analysis of its expression in different tissues, salt and drought stresses, and different phytohormone treatments at different time points. Additionally, H3 and H4 acetylation were analyzed under salt and drought stress. Taken together, this study comprehensively explored and analyzed the functions of the GmHAT and GmHDAC families, with a view to laying a solid foundation for subsequent studies of their functions.

## Materials and methods

### Plant material and treatment

Soybean material was grown under conditions as previously described (Sun et al., 2019). Full and consistent growing Williams 82 seeds were sterilized using 70% alcohol for 1 minute and then rinsed with distilled water. Sterilized soybean seeds were germinated on moist filter paper. Four well germinated seeds were then selected and sown in pots containing vermiculite. All seedlings were grown under a photoperiod of 16 h/8 hours (light/dark) and a temperature of 25 °C/20 °C (light/dark) and were regularly watered with Hoagland’s liquid medium. For 2-week-old seedlings, 200 mL of 200 mM NaCl solution and 10% PEG6000 solution were added to simulate salt and drought stress treatments, respectively. Based on previous studies (Chen et al., 2014; Lv et al., 2022; Zhang et al., 2025), leaf tissue was collected at 0, 6, 12, and 24 hours post-treatment. Three biological replicates were prepared at each time point, with each replicate comprising at least three seedling pots. The 0 hour sample served as the untreated control group. All samples were immediately placed in liquid nitrogen upon collection and stored at -80 °C for subsequent RT-qPCR analysis of gene expression patterns.

For hormone treatments refer to previous descriptions (Xiong et al., 2024). In summary, this study treated 2-week-old soybean seedlings with five hormones at a concentration of 100 μmol/L (abscisic acid: ABA, indole-3-acetic acid: IAA, gibberellin: GA, methyl jasmonate: MeJA, and salicylic acid: SA), with samples collected at 0, 6, 12, and 24 hours post-treatment. Samples collected at 0 hour served as untreated controls. Changes in gene expression patterns following hormone treatment were analyzed at 6, 12, and 24 hours. Sampled leaves were rapidly placed in liquid nitrogen and stored at - 80 °C for subsequent RT-qPCR. There were at least three pots of seedlings per treatment time point and at least three biological replicates per treatment time point.

### RNA isolation and RT-qPCR

Total RNA from soybean leaves was extracted using the SteadyPure Plant RNA Extraction Kit (AG21019, Accurate Biology, China) according to the manufacturer’s instructions. First-strand cDNA was synthesized using UEIris RT mix with DNase (All-in-One) (R2020S, UElandy, China). To analyze the expression patterns of all genes, RT-qPCR analysis was performed in a CFX ConnectTM real-time system (BIO-RAD, USA) using Universal SYBR Green Super Mix (S2024S, UElandy, China). Data were normalized using *Actin11* as an internal control and relative gene expression was calculated using the 2^-ΔΔCT^ method. The experiment was conducted using three independent biological replicates, with three technical replicates for each sample. Gene-specific primers for RT-qPCR are listed in **Supplementary Table 1**.

### Histone extraction and western blotting

Isolated histones were extracted using EpiQuik Total Histone Extraction Kit (Epigentek, Farmingdale, NY, USA, cod. OP-0006) according to the instructions. Briefly, leaves of soybean seedlings treated with salt and drought stress for 12 hours were ground into powder, resuspended with 1 mL of diluted prelysis buffer (1×), and incubated with ice bath agitation for 10 minutes to remove the plasma membrane. after centrifugation at 4°C, 9391× *g* for 1 minutes, the precipitate was resuspended in 50 μL of lysis buffer and incubated for 30 minutes on ice. The lysate was centrifuged at 4°C, 13523× *g* for 5 minutes and the supernatant was transferred to a new tube. To prevent protein aggregates from interfering with histone extraction, 0.3 volume of DTT-free equilibration buffer was immediately added to each sample for western blotting analysis.

Western blotting was performed as previously described (Liu et al., 2023). Proteins were separated on a 12% sodium dodecyl sulfate‐polyacrylamide gel and transferred onto a polyvinylidene difluoride membrane (Millipore, Germany). The following primary antibodies were used: anti‐H3 (ab1791; Abcam, UK), anti‐H3ac (06‐599; Millipore, Corp, USA), anti-H3K9ac (ab10812; Abcam, UK), anti-H3K18ac (ab1191; Abcam, UK), anti‐H4ac (ab177790; Abcam, UK), anti-H4K5ac (ab51997; Abcam, UK), anti-H4K8ac (ab45166; Abcam, UK). Based on previous research findings, the above acetylated antibodies are expressed in plants in response to salt stress or drought stress (Liu et al., 2023; Xue et al., 2024; Yung et al., 2022). Histone H3 was used as a loading control. Signals were detected using the SuperSignal West Pico Plus chemiluminescent substrate (Thermo Scientific; Vazyme, China).

### Genome-wide identification of GmHATs and GmHDACs

To identify members of the soybean GmHAT and GmHDAC families, protein sequences of the *A.thaliana* and *O.sativa* HATs and HDACs genes were downloaded from the TAIR database (https://www.arabidopsis.org) and the Rice Genome Annotation Project (https://rice.uga.edu/), respectively. The protein sequences of HATs and HDACs from Arabidopsis and rice, respectively, were used as input sequences to search the entire soybean genome (Wm82.a6.v1) through the BLASTP (https://phytozome-next.jgi.doe.gov/blast-search) program in the Phytozome 13 database using the default parameters. Next, based on the previous research methodology (Du et al., 2022; Guo et al., 2024), the following entries were downloaded from the Pfam database: (GNAT: PF00583, CBP: PF08214, TAFII250: PF09247, MYST: PF01853; RPD3/HDA1: PF00850, SIR2: PF02146, HD2: PF17800). We employed the same strategy to search for conserved domains in putative GmHATs and GmHDACs, ensuring they belong to the HAT and HDAC families. Finally, verification was performed using the CD-Search database (https://www.ncbi.nlm.nih.gov/Structure/cdd/wrpsb.cgi) and the SMART tool (https://smart.embl.de/smart/change_mode.cgi).

Chromosomal positions and amino acid numbers of the genes were obtained using gff annotation files obtained from Phytozome 13. The basic physicochemical properties of GmHATs and GmHDACs, including theoretical isoelectric point (pI), molecular weight and instability coefficient, were analyzed using ExPASy (https://www.expasy.org/). The online software Plant-mPLoc (http://www.csbio.sjtu.edu.cn/bioinf/plant-multi/) was used to predict the subcellular localization of GmHATs and GmHDACs.

### Phylogenetic, gene structure, conserved motif and domain analysi*s*

To classify the GmHATs and GmHDAC, protein sequence comparison was performed using mafft (Katoh et al., 2005) based on the pre-downloaded protein sequences of *O*.*sativa*, *A*.*thaliana* and *G.max*. The evolutionary tree is generated using fasttree (Price et al., 2009) and finally visualized using the R package ggtree (Yu et al., 2017). Gene structure mapping based on gff annotation files using the R package transPlotR. The conserved motifs of the genes were analyzed using the online database MEME (http://meme-suite.org/tools/meme) with the maximum conserved motif search value set to 20 and other parameters defaulted. The motif distribution maps were generated by TBtools software using an XML file containing motif pattern information obtained from MEME software. Conserved structural domains of genes were visualized using TBtools (Chen et al., 2020a) based on CD-Search database (https://www.ncbi.nlm.nih.gov/Structure/cdd/wrpsb.cgi) results.

### Chromosomal distribution and colinearity analysis of genes

The *GmHATs* and *GmHDACs* were localized to chromosomes using TBtools based on *G.max* genome and annotation file data. Tandem and fragment duplication events of GmHATs and GmHDACs were analyzed using Multiple Covariance Scanning Kit X (MCScanX) (Zhu et al., 2014), using default parameters. The multiple synteny plot tool in Circos tool and TBtools was used to visualize the covariance between members of the *G.max* GmHATs and GmHDAC families and other species (dicots: *A. thaliana* and *C. arietinum*; monocots: *Z. mays* and *O. sativa*).

### *Cis-elements* and protein interaction network analysis

Sequences 2000 bp upstream of the transcription start site were extracted as promoter sequences. Sequences were submitted to the online database PlantCARE (Lescot et al., 2002) (http://bioinformatics.psb.ugent.be/webtools/plantcare/html/) to analyze potential *cis-elements* in the promoter region. The analysis results were visualized using TBtools. Protein interaction networks for GmHATs and GmHDACs were predicted using the STRING (https://cn.string-db.org/) database. The sequences of all proteins were used as a query, with *G.max* selected for species and other parameters defaulted. Subsequently, protein interaction networks were exported and visualized using Cytoscape (version 3.9.1).

### Tissue expression patterns of *GmHATs* and *GmHDACs*

Download raw RNA-seq data from NCBI for different tissues of Williams 82 varieties that have been publicly reported (stem: PRJDB7219; leaf, root: PRJNA407016; seedling: PRJNA525277; flower, pod: PRJNA236472; Seed: PRJNA677883; Cotyledon: PRJNA262564). The data were aligned to the *G.max* genome (Wm82.a6.v1) using hisat2 software, and the transcripts per kilobase million (TPM) values of the genes were further calculated using stringtie and visualized using TBtools.

## Results

### Identification of GmHAT and GmHDAC families in soybean genome

We used *A.thaliana* and *O.sativa* HAT and HDAC protein sequences on the basis of the Phytozome 13 BLAST program as input sequences to search in the *G.max* (Wm82.a6.v1) genome. After identification of the conserved structural domains of HAT and HDAC, a total of 12 *GmHATs* and 28 *GmHDACs* were identified. We named the subfamilies according to their position on the chromosome, and analyzed their protein molecular weights, theoretical isoelectric points (pI), subcellular localization, and other physicochemical properties (**Supplementary Table 2**).

Of the 12 GmHATs, the amino acid numbers ranged from 434 aa (GmHAM1) to 1890 aa (GmHAF2), and the molecular weights ranged from 50.10 kDa (GmHAM2) to 214.56 kDa (GmHAF2). The smallest pI was 5.22 (GmHAG3) and the largest was 8.45 (GmHAC1). Interestingly, 12 GmHAT proteins were classified as unstable, with instability coefficients all exceeding 40, necessitating careful handling *in vitro*. Concerning 28 GmHDAC proteins, the amino acid numbers ranged from 114 aa (GmHDA10) to 656 aa (GmHDA5) and the molecular weights ranged from 13.01 kDa (GmHDA10) to 73.05 kDa (GmHDA5). The smallest pI was 4.61 (GmHDT6) and the largest was 9.6 (GmHDA10), with the majority (22 of 28) having a pI < 7, suggesting that the GmHDAC gene family favors acidic amino acids. The instability coefficients ranged from 24.39 (GmHDA14) to 52.1 (GmHDT1). Predictive analysis of subcellular localization showed that soybean GmHAT and GmHDAC exhibited diverse distribution patterns. Among them, 10 GmHAT proteins were mainly localized in the nucleus; 3 SIR2 subfamily members were specifically localized in chloroplasts; whereas GmSRT3 showed chloroplast and cytoplasmic co-localization patterns. Notably, 2 GmHAT and 8 GmHDAC belong to the nucleoplasmic co-localization, suggesting that 10 genes may catalyze both histone acetylation and non-histone acetylation. Taken together, the soybean GmHAT and GmHDAC family members showed significant diversity in physicochemical properties and subcellular localization, suggesting that they may be functionally differentiated in epigenetic regulation and participate in the dynamic balance of histone and non-histone acetylation/deacetylation through organelle-specific localization.

### Phylogenetic analyses, gene structure, conserved motifs, domain of GmHATs and GmHDACs

**Figure 1:**
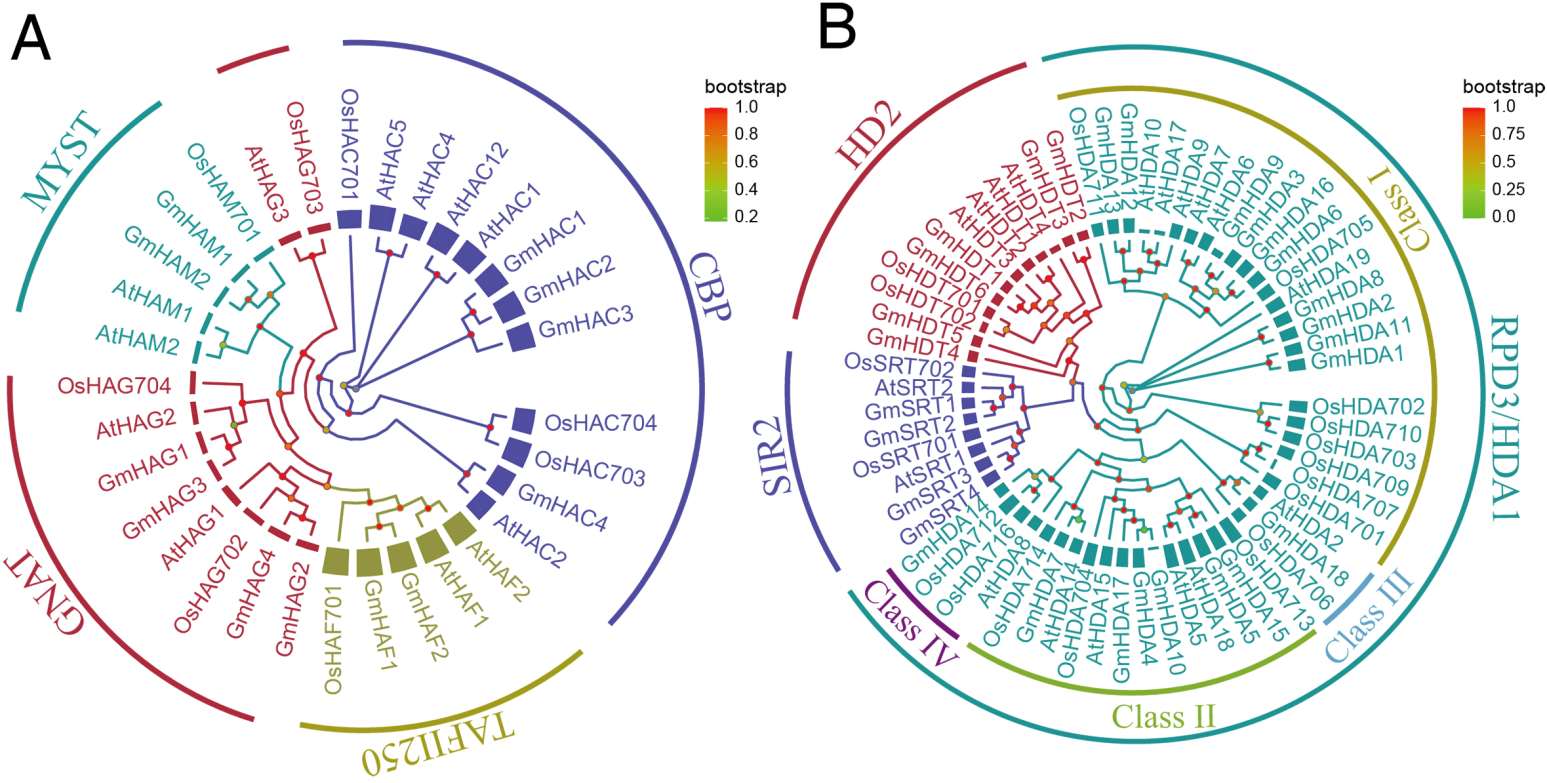
Phylogenetic analysis of homologous proteins of *O.sativa, A.thaliana*, and *G.max* HATs and HDACs. **(A)** and **(B)** indicate HATs and HDACs, respectively, and squares represent the number of amino acids. Os, At, and Gm indicate *O.sativa, A.thaliana*, and *G.max* respectively.

Based on the protein sequences and classification of *A.thaliana* and *O.sativa* HATs and HDACs, we performed phylogenetic analyses of soybean GmHATs and GmHDACs using the neighbor-joining method (**Figure 1**). The results indicated that the 12 GmHAT members were categorized into four subfamilies, with the GNAT and CBP subfamilies containing 4 members each, and the TAFII250 and MYST subfamilies 2 members each. The 28 GmHDACs were mainly categorized into 3 subfamilies, with 18 members of the RPD3/HDA1 subfamily, 4 members of the SIR2 subfamily, and 6 members of the HD2 subfamily. Among the 18 RPD3/HDA1 subfamily members, Class I had 10 members, Class II had 6 members, and Class III and Class IV each had 1 member.

**Figure 2:**
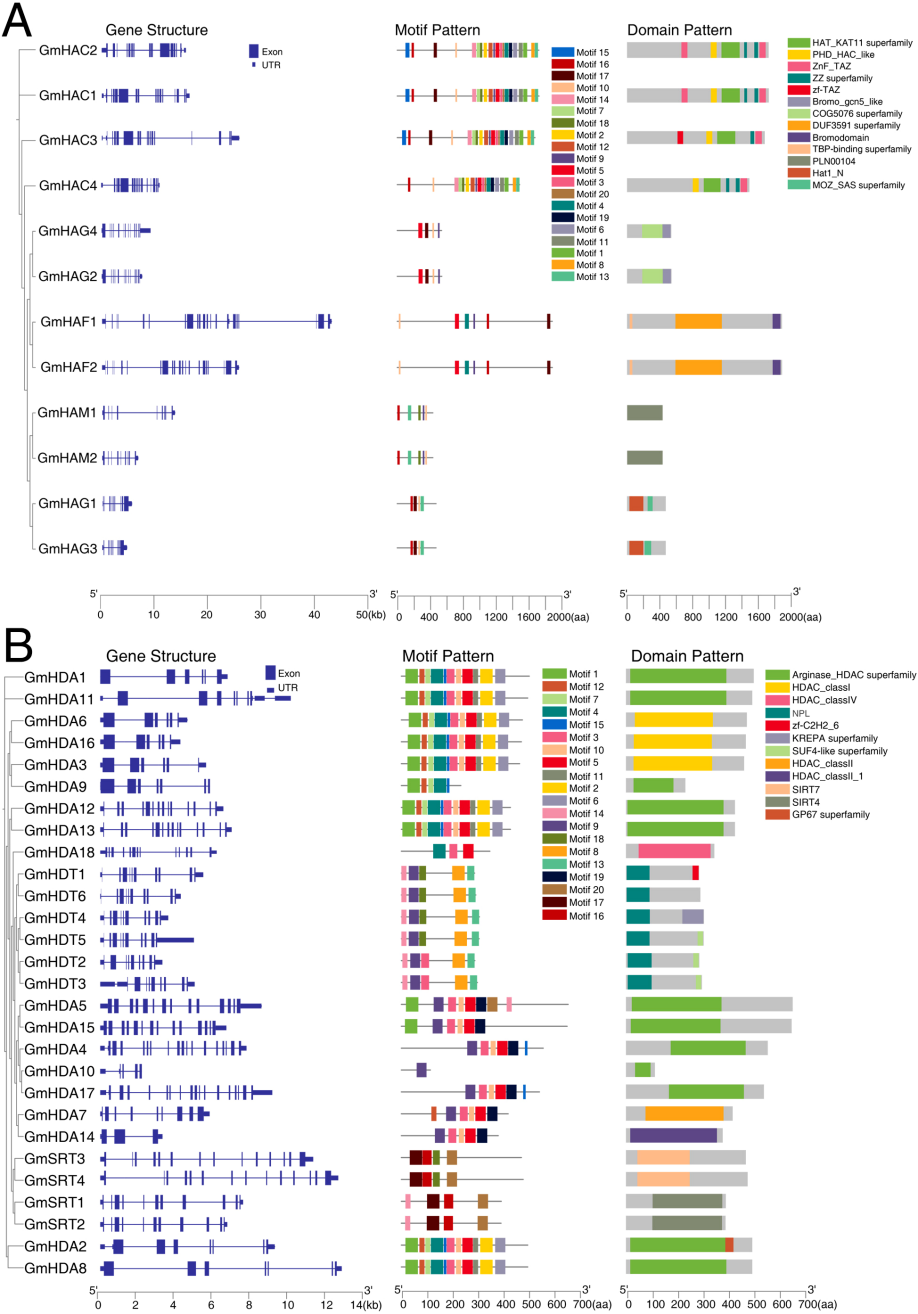
Gene structure, motif and domains of GmHAT **(A)** and GmHDAC **(B)**. The phylogenetic trees, motif patterns and structural domain molds of GmHAT and GmHDAC are shown from left to right.

Analysis of the phylogenetic tree for the GmHAT and GmHDAC families showed results consistent with **Figure 1** (**Figure 2**). To analyze the structural composition of the *GmHATs* and *GmHDACs*, we constructed a structural map based on the soybean genome sequence and annotation files, including untranslated regions (UTRs), exons and introns (**Figure 2**). Results showed that all genes contained UTRs, with great variation in the number of exons and introns. The number of *GmHATs* exons ranged from 9 to 21 and the number of introns ranged from 9 to 20. While *GmHDACs* exons are between 3 and 17 and introns are between 2 and 17. We identified GmHAT and GmHDAC proteins through an online server (MEME). A total of 20 motifs were identified in 12 GmHATs and showed great variation (**Figure 2**). For GmHATs, harboring motifs ranged from 4 to 20. For these GmHATs, a total of 13 conserved structural domains were identified, and the number of GmHATs structural domains ranged from 1 to 6. The GNAT subfamily genes contain the Hat1_N and Bromo_gcn5_like structural domains; the CBP subfamily genes all contain the HAT_KAT11_superfamily structural domain; the MYST subfamily genes all contain the PLN00104 structural domain; and the TAFII250 subfamily all contain the Bromodomain. The motifs of the 28 GmHDACs proteins varied significantly, and a total of 20 were identified (**Figure 2**). The motifs of GmHDACs proteins range from 1 to 11. Only one motif was present in GmHDA10, whereas both GmSIR2 subfamilies contained motif 17 and motif 20. Conserved structural domain analysis revealed that both RPD3/HDA1 contain HDAC structural domains; NPL structural domains are present only in the plant-specific GmHD2 subfamily; and SIRT4 and SIRT7 are present only in the GmSIR2 subfamily. This result is highly consistent with the phylogenetic analysis.

### Chromosome localization, colinearity analysis of GmHATs and GmHDACs

**Figure 3:**
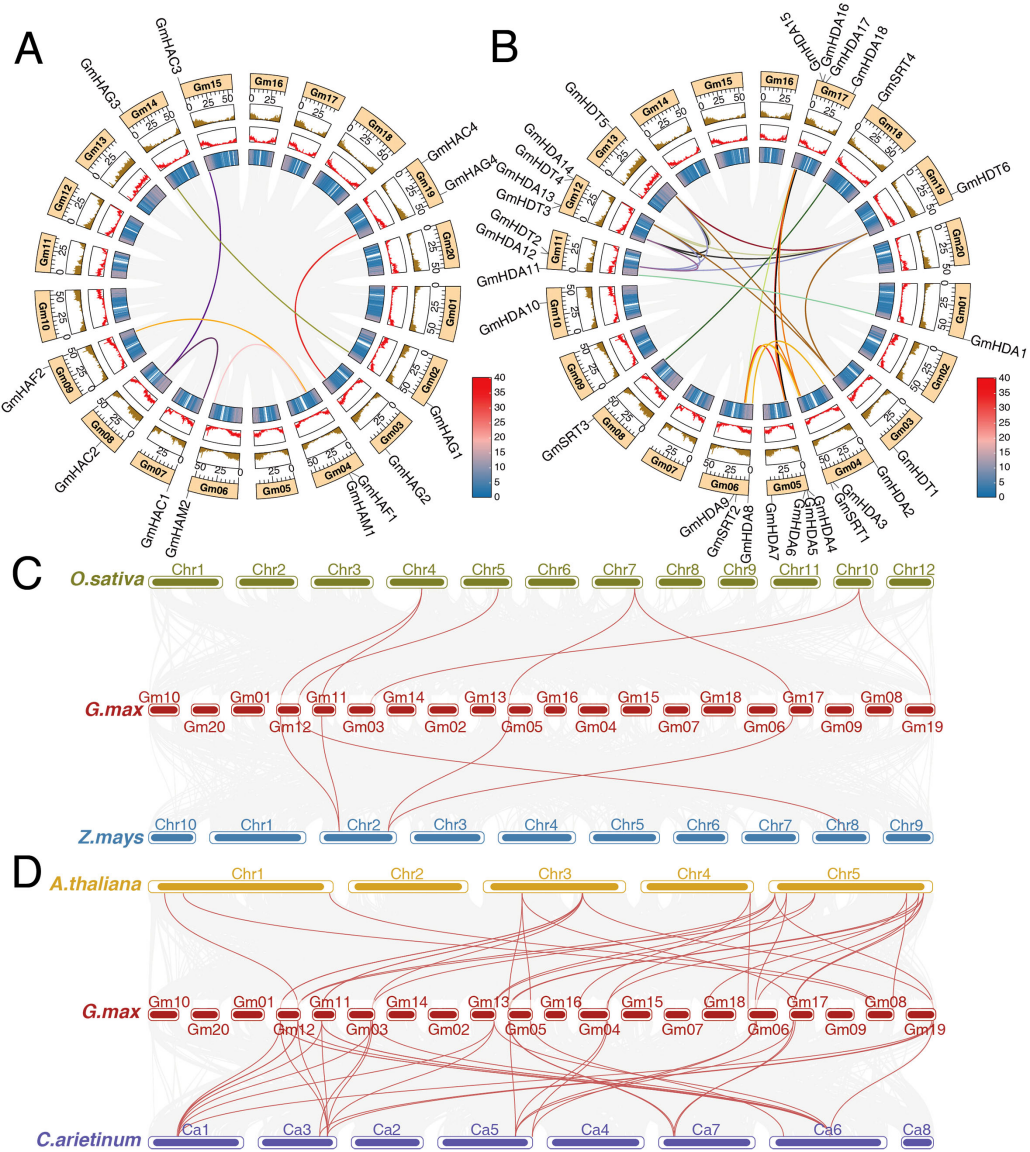
Intraspecific and interspecific colinearity analysis of HDAC and HAT. **(A)** and **(B)** indicate the intraspecific colinearity of GmHATs and GmHDACs, respectively. **(C)** indicates interspecies colinearity analysis of *G.max* GmHATs and GmHDACs with the monocotyledonous plants *O.sativa* and *Z.mays*. **(D)** Indicates the analysis of interspecific colinearity of *G.max* GmHATs and GmHDACs with dicotyledonous plants *A.thaliana* and *C.arietinum*.

Using the soybean genome and annotation files, we analyzed the positions of 12 *GmHATs* and 28 *GmHDACs* on the chromosomes, with all 40 members displayed in specific positions (**Figure 3**; **Supplementary Figure 1**). The 40 genes were unevenly distributed across the eighteen chromosomes (except for Gm16 and Gm20), and genes of the same subfamily were randomly distributed across the chromosomes. Gm01, Gm02, Gm09, Gm10, Gm13, Gm14, Gm15, and Gm18 contain 1 gene each; Gm03 and Gm08 contain 2 genes; Gm11 and Gm19 contain 3 genes each; Gm05, Gm06, Gm12, and Gm17 contain 4 genes each; Gm04 contains 5 genes. Above results suggest that *GmHATs* and *GmHDACs* may play important roles in the complex life processes of soybean.

Tandem duplication is a major driver of gene family expansion in plants, and chromosomal regions in the 200-kb range containing two or more genes are defined as tandem duplication events (Chen et al., 2017). A total of 2 tandem repeat events were observed in this study, including *GmHDA12* and *GmHDT2*, and *GmHDA13* and *GmHDT3* (**Supplementary Figure 1**). Intra-species colinearity analysis revealed 11 segmental repeat events for *GmHAC1*, *GmHAC2*, *GmHAC3*, *GmHAG1*, *GmHAG2*, *GmHAG3*, *GmHAG4*, *GmHAF1*, *GmHAF2*, *GmHAM1*, and *GmHAM2*. Among them, *GmHAC1*, *GmHAC2* and *GmHAC3* showed correlation (**Figure 3A**). GmHDACs exhibited 25 fragment repeat events. Among them, GmHDA3, GmHDA9, GmHDA6, and GmHDA16 showed linear correlation; *GmHDT1*, *GmHDT4*, *GmHDT5*, and *GmHDT6* showed linear correlation; *GmHDT2*, *GmHDT4*, *GmHDT5*, and *GmHDT6* showed linear correlation; *GmHDT3*, *GmHDT4*, and *GmHDT5*, *GmHDT6* showed linear correlation (**Figure 3B**). To further understand gene duplication events between *GmHATs* and *GmHDACs* species, we compared the homology of soybean with *A.thaliana* and *C.aruetunum* (both dicots) (**Figure 3C**), and *O.sativa* and *Z.mays* (both monocots) (**Figure 3D**). The results showed a higher degree of colinearity between the soybean and dicots genomes compared to the monocots plants. Additionally, we observed that most *A.thaliana* and *C.aruetunum HATs* and *HDACs* have corresponding direct homologs in soybean, with many genes having more than two direct homologs. The presence of orthologous gene pairs indicates conservation of gene function and the potential for similar biological processes between these species.

### *Cis-element* analysis of the GmHAT and GmHDAC promoters

**Figure 4:**
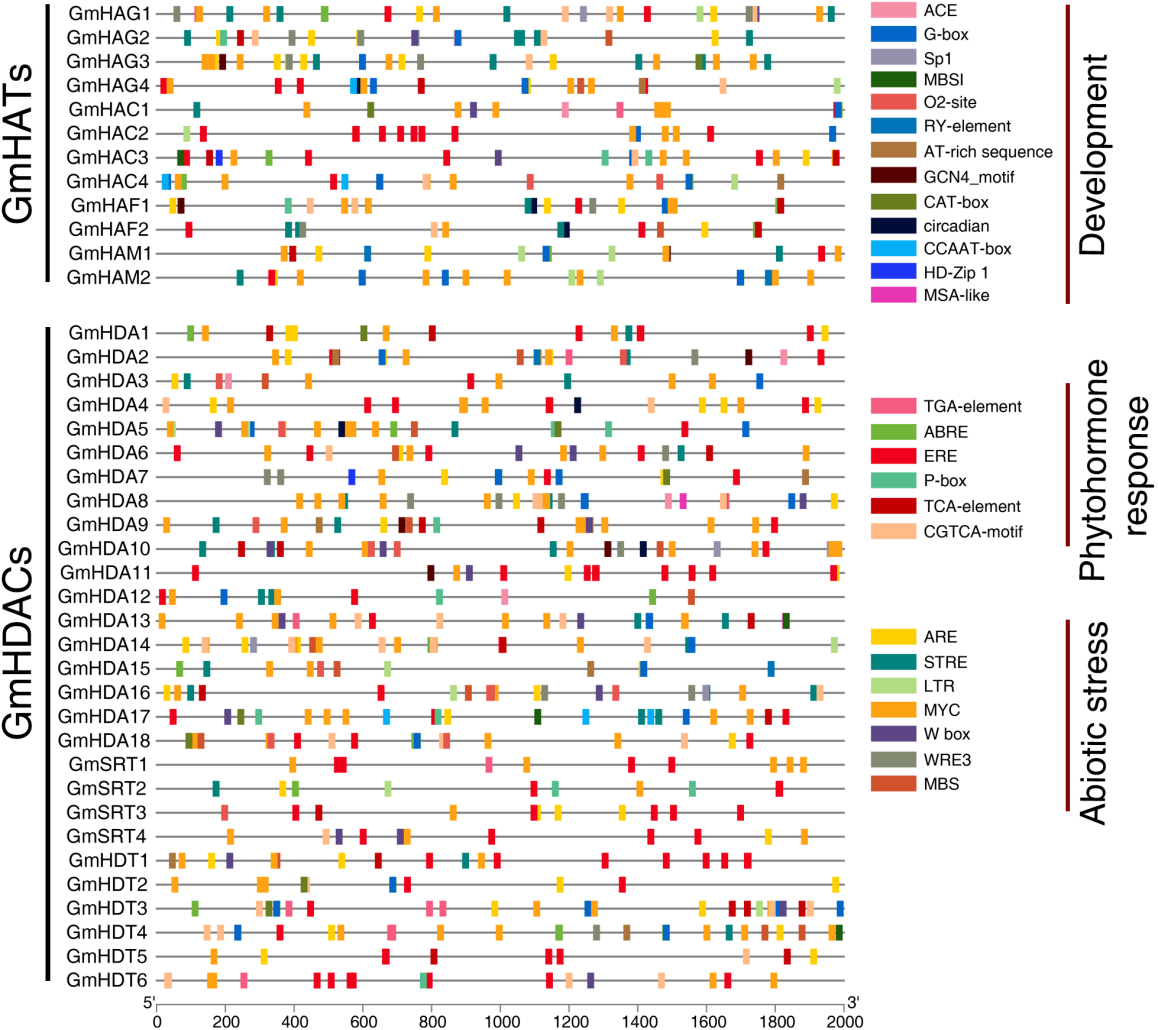
*Cis-elements* in the promoters of *GmHAT* and *GmHDAC*. Lines represent sequences 2kb upstream of the gene transcription start site, and different colored rectangles represent different response elements. These elements are categorized into three functional groups: Development, Phytohormone response, and Abiotic stress. All three types of elements are distributed within the promoter regions of the *GmHAT* and *GmHDAC* families.

To investigate the potential regulatory mechanisms of *GmHATs* and *GmHDACs* during soybean development, we extracted the promoter sequence 2.0 kb upstream of the transcription start site and analyzed its *cis-element* using the PlantCARE website (**Figure 4**). The results show that common *cis-element* in promoter and enhancer regions such as TATA-box and CAAT-box are still the most widely distributed. Then, we selected 26 *cis-element* related to plant development, phytohormone response and abiotic stress. Light responsive elements and Low-temperature responsiveness (LTR) are present in all *GmHATs* and *GmHDACs* (**Figure 4**; **Supplementary Figure 2**). *GmHATs* all contain Abscisic acid responsiveness (ABRE). For *GmHDACs*, all but *GmHDA8* and *GmHDA15* contained more than one Ethylene response element (ERE). In addition, results indicated that the promoters of *GmHATs* and *GmHDACs* contain multiple stress-responsive elements, such as Drought-inducibility (MBS), Low-temperature responsiveness (LTR), Wound-responsive element (WRE3), Anaerobic induction (ARE), and Defense and stress responsiveness (STRE) (**Figure 4**; **Supplementary Figure 2**). Taken together, *GmHATs* and *GmHDACs* may play positive or negative regulatory roles in different abiotic stresses.

### Interaction network analysis of GmHATs and GmHDACs

?>Interaction network analysis provides an effective way to analyze the biological functions of proteins and reveal their molecular mechanisms. Therefore, we predicted the interacting proteins of GmHAT and GmHDAC using the STRING database. A total of 17 nodes were identified in GmHATs, and 61 interacting proteins were observed in these nodes (**Supplementary Figure 3A**; **Supplementary Table 3**). Whereas a total of 30 nodes were identified in GmHDACs, 224 interacting proteins were observed in these nodes (**Supplementary Figure 3B**; **Supplementary Table 3**). Non-interaction of CBP and TAFII250 subfamily members in the GmHAT family. However, GmHATs interacted with C2 domain-containing proteins (I1N3I5, A0A0R0J7I7), Homeobox domain-containing proteins (I1LMP3, I1JI62) and Histone deacetylase domain-containing protein (I1MXC3). For GmHDACs, the HD2 subfamily (except for GmHDT4) did not interact with either of the other two subfamilies. Possible interaction between SIR2 and RPD3/HDA1 subfamily members and possible interaction with pathogenesis-related homeodomain protein (A0A368UH09, K7LC20, K7MK45, I1LVM1), HhH-GPD domain-containing protein (K7LYE7) and HhH-GPD domain-containing protein.

### Expression analysis of *GmHAT* and *GmHDAC* in different tissues

**Figure 5:**
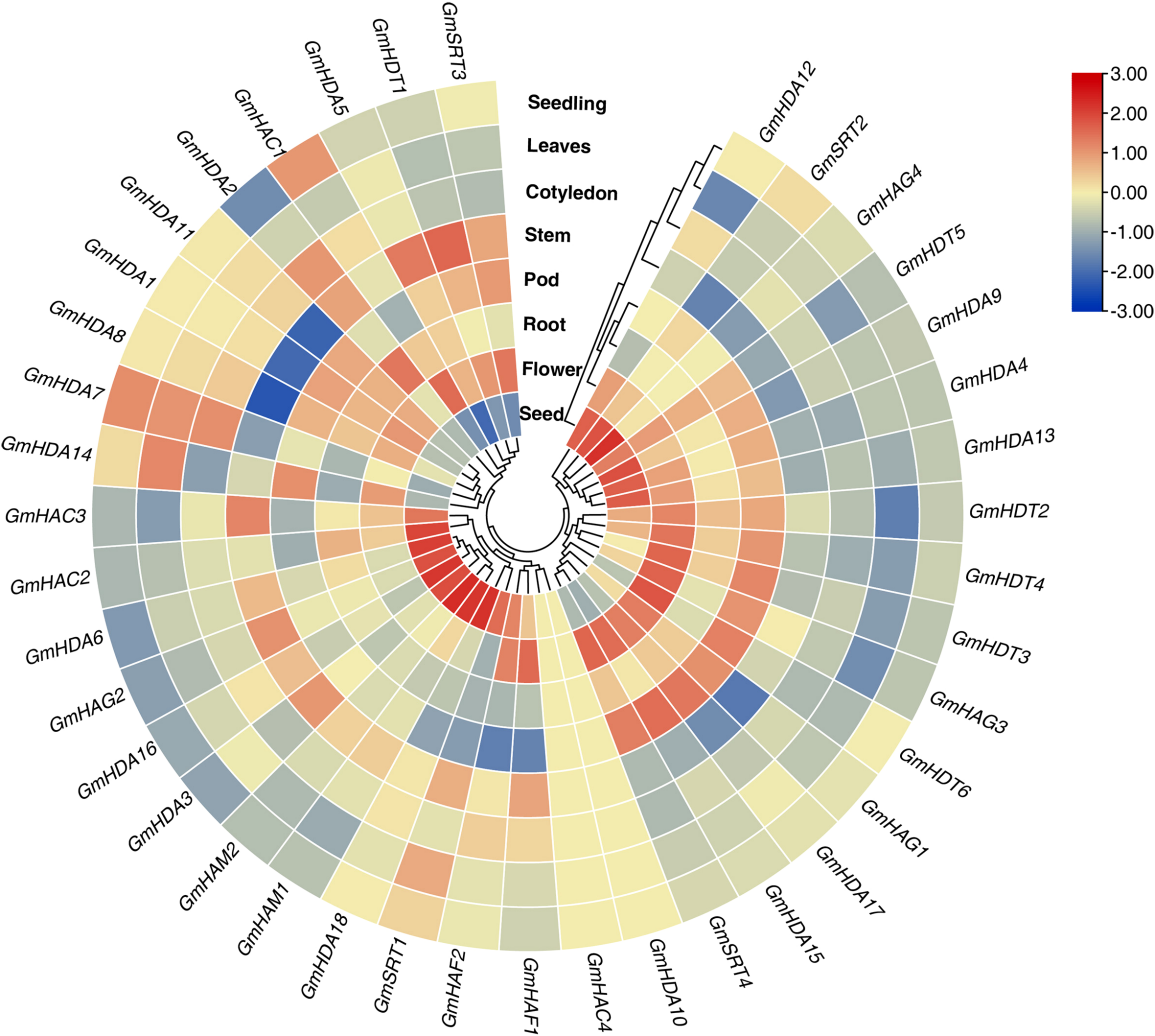
Heatmap of expression levels of *GmHAT* and *GmHDAC* in different tissues. This circular heatmap displays gene expression patterns across eight distinct tissues: seedlings, leaves, cotyledons, stems, pods, roots, flowers, and seeds. Gene expression levels (Transcripts Per Million, TPM) underwent Log2 transformation followed by row-scale normalization. The heatmap color transitions from blue (low expression) to red (high expression), with values representing the Z-score of relative expression levels. The concentric rings represent different tissue types from inner to outer layers.

To further understand the functions of *GmHAT* and *GmHDAC*, we analyzed their expression levels in seedling, leaves, cotyledon, stem, pod, root, flower, and seed (**Figure 5**). The results showed that *GmHATs* and *GmHDACs* existed widely expressed and the expression levels were highly variable in different tissues. Among the 12 *GmHATs* and 28 *GmHDACs*, expression levels of 38 genes were detected in at least one tissue. In contrast, expression levels of *GmHAC4* and *GmHDA10* were not detected in any of the tissues, and these genes may be pseudogenes or have unique expression patterns not detected in our study. Some genes (*HAC1/2/3*, *HAF1/2*) were not differentially expressed in the tissues detected. However, many genes exhibit tissue-specific expression patterns. For instance, *GmHAG2*, *GmHAG4*, and *GmHAM2* had the highest expression in seeds, and *GmHDT1* had the highest expression in stem. *GmHDT5* had the highest expression in flower. The differential gene expression levels among different tissues provide an important basis for screening candidate genes with more potential for *in vivo* studies.

### Expression patterns of *GmHAT* and *GmHDAC* under salt and drought stresses

**Figure 6:**
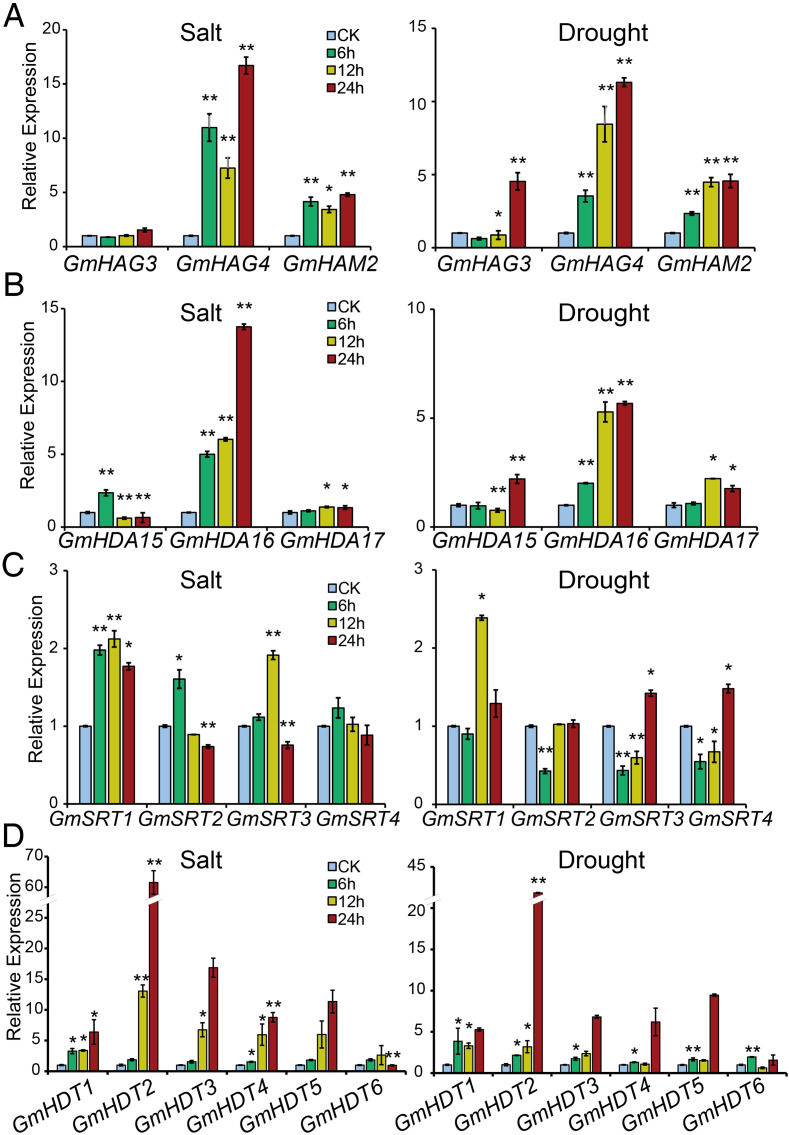
Expression profiles of 16 *GmHATs* and *GmHDACs* in soybean seedlings under salt and drought stress. **(A–D)** Relative expression patterns of genes from the GmHAT family **(A)**, RPD3/HDA1 subfamily **(B)**, SIR2 subfamily **(C)**, and HD2 subfamily **(D)** at different time points. The Y-axis represents the relative fold change in expression levels, normalized using *Actin11* as the reference gene and calibrated against the untreated control group (CK, set to 1). Statistical analysis using *t*-test (* indicates *p* < 0.05, ** indicates *p* < 0.01).

The promoter regions of *GmHAT* and *GmHDAC* contain a large number of stress-related homeostatic regulatory elements, suggesting that they may play important roles in soybean adversity stress response (**Figure 4**). In this study, we used salt and drought stress as examples and randomly selected 16 genes out of 28 for analysis (**Figure 6**). The results indicated that in *GmHATs*, both *GmHAG4* and *GmHAM2* were significantly induced to be expressed by both salt and drought stresses (**Figure 6A**). *GmHAG3* was not significantly induced by salt stress, but was significantly induced by drought treatment at 12 hours and 24 hours (**Figure 6A**). For *GmHDACs*, both salt and drought significantly induced the expression of the RPD3/HDA1 subfamily gene *GmHDA16*. At 12 hours and 24 hours post-stress treatment, *GmHDA17* expression was also significantly induced. Conversely, *GmHDA15* expression was significantly suppressed at 12 hours and 24 hours under salt treatment and at 12 hours under drought treatment (**Figure 6B**). Compared with other genes, the overall expression levels of SIR2 subfamily genes were low (**Figure 6C**). *GmSRT1* expression was significantly induced by salt stress, while drought treatment alone induced it significantly after 12 hours. *GmSRT2* showed opposite responses to salt and drought treatments at 6 hours: salt significantly induced its expression, while drought significantly suppressed it. *GmSRT3* and *GmSRT4* were significantly induced at 6 hours and 12 hours under salt and drought treatments. Meanwhile, *GmSRT3* was significantly induced at 12 hours and inhibited at 24 hours under salt stress, while *GmSRT4* showed no significant changes in response to salt stress (**Figure 6C**). The expression of other HD2 genes, except for *GmHDT6*, was induced up-regulated to varying degrees with treatment time (**Figure 6D**). Notably, the HD2 subfamily gene, *GmHDT2*, was significantly induced to be expressed by 24 hours of salt stress treatment, which was 61-fold higher than that of normal conditions, whereas drought stress treatment for 24 h up-regulated its expression by 43-fold.

**Figure 7:**
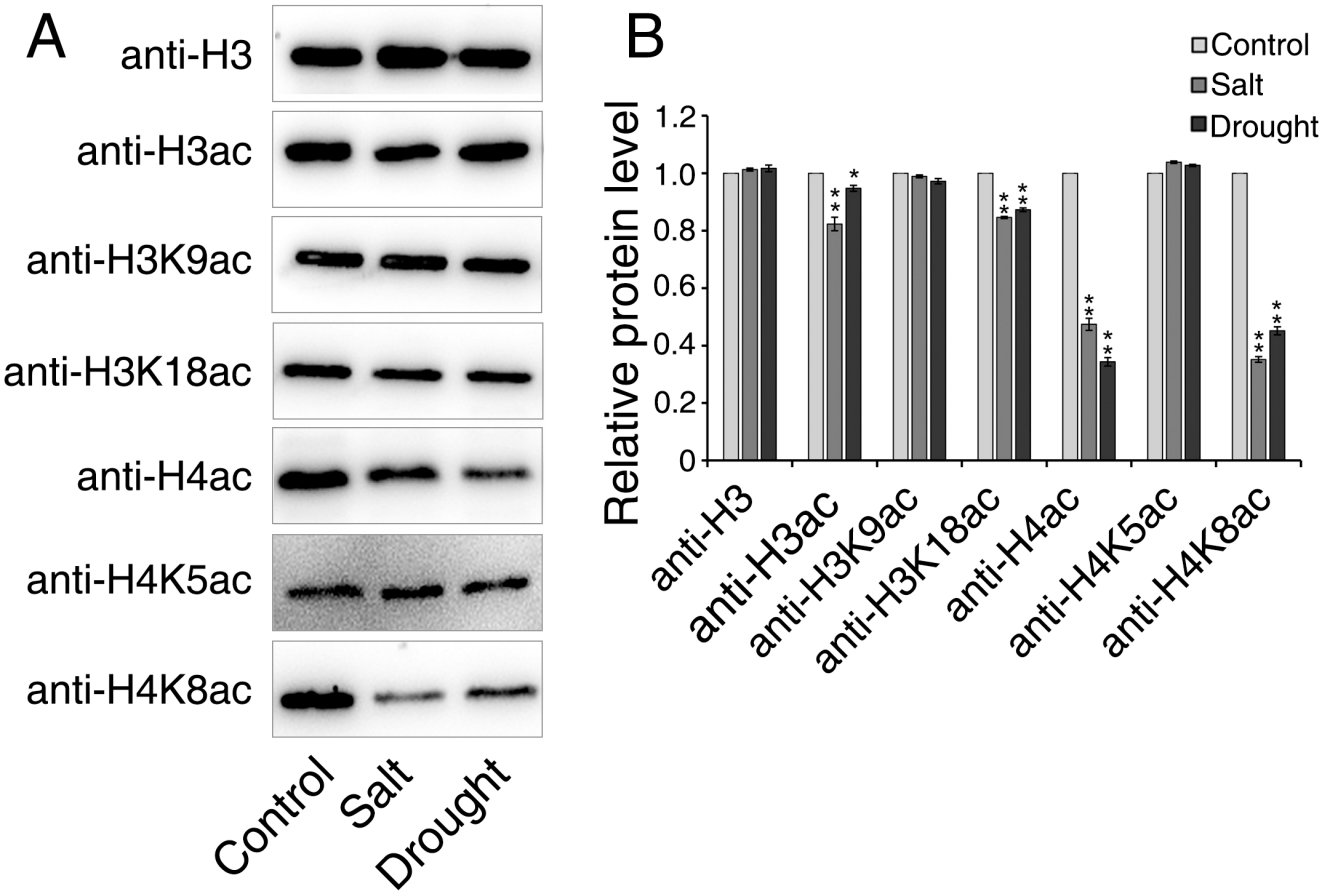
Histone H3 and H4 acetylation levels under salt and drought treatments. **(A)** Immunoblot analysis of H3 and H4 acetylation levels in soybean leaves under salt and drought stress. Histones were separated by SDS-PAGE and subjected to western blotting using the antibodies shown on the left. Anti-H3 was used as a control. **(B)** Statistical analysis of **(A)** protein blotting results. Normalized to the loading of H3. Quantitative analysis of immunoblotting signals relative to the control (set as 1) was performed using ImageJ software. Each value represents the mean ± standard deviation (SD) of three biological replicates. Significant differences between the treatment group and the control group (* indicates *p* < 0.05, ** indicates *p* < 0.01, two-tailed Student's t-test).

The expression profiles of *GmHAT* and *GmHDAC* were significantly changed under salt and drought stress, so we further analyzed the histone acetylation patterns of soybean seedlings in response to salt and drought stress. We analyzed histones extracted from soybean seedlings treated with salt and drought stress for 12 hours by immunoblotting. The results showed that both salt and drought treatments resulted in a significant reduction in histone H3 and H4 acetylation levels (**Figures 7A, B**). Among these, H3K9ac showed no significant changes before and after stress treatments, but H3K18ac decreased markedly following salt and drought treatments. H4 acetylation analysis revealed no change in H4K5ac, but H4K8ac exhibited significant alterations following both stress treatments (**Figures 7A, B**). Interestingly, reduced acetylation showed a negative correlation with the high expression of HD2 family genes, particularly *GmHDT2*, *GmHDT3*, *GmHDT4*, and *GmHDT5*. Previous reports indicated that Arabidopsis RNAi-mediated HD2 family gene *HDT1* (*AtHD2A*) leads to loss of H3K9 deacetylation (Lawrence et al., 2004). Overexpression of the HD2 family gene *OsHDT701* in rice resulted in reduced H4K5/K16 acetylation levels (Ding et al., 2012). During drought stress response, HD2A and HD2B negatively regulate ABA biosynthesis by deacetylating H4K5ac on the key ABA synthesis gene *NCED9*, thereby suppressing its expression (Han et al., 2023). We therefore hypothesize that reduced acetylation levels, particularly at the H4 site, may be regulated by the aforementioned HD2 family genes, though this requires further validation.

### Expression patterns of *GmHAT* and *GmHDAC* in response to hormone treatment

**Figure 8:**
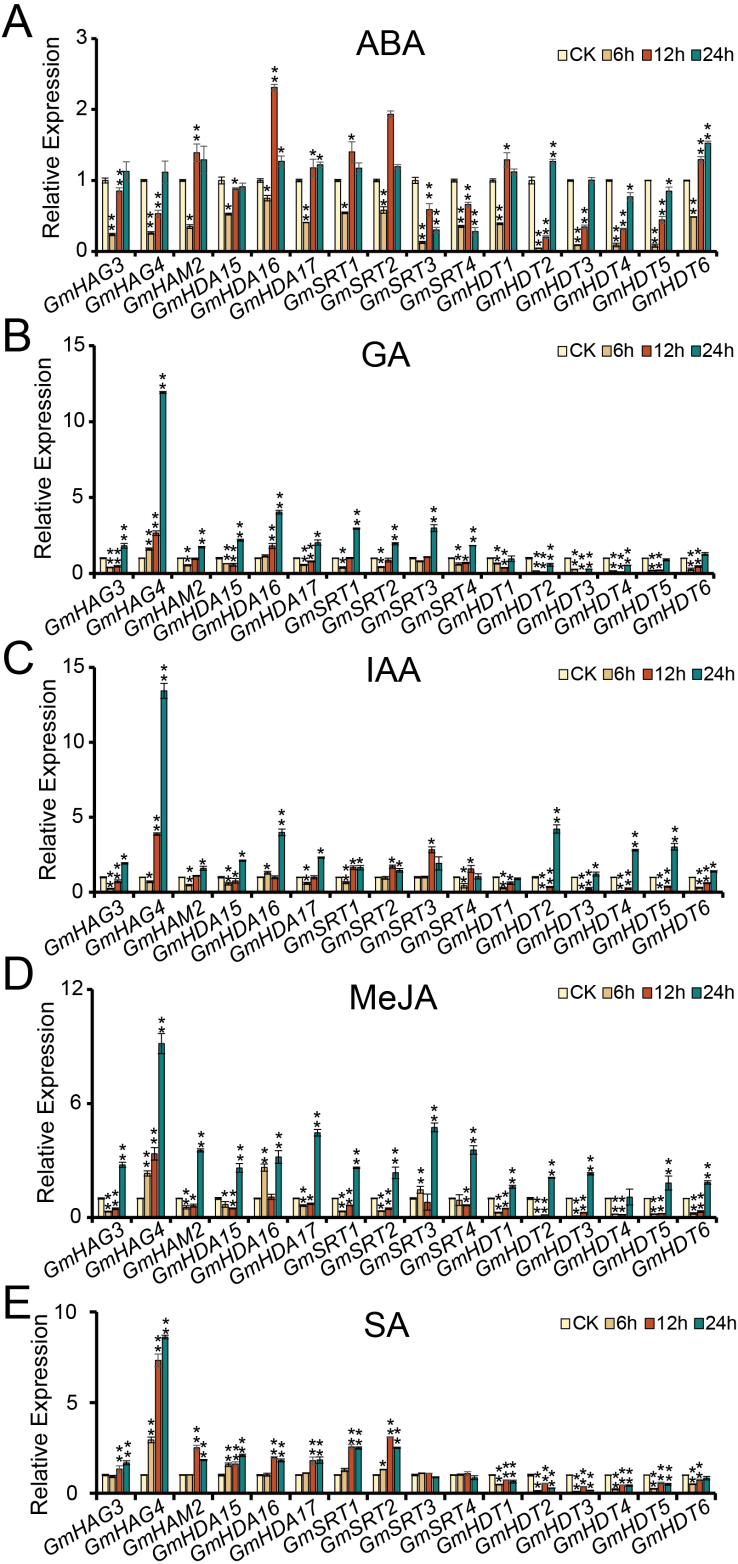
Expression profiles of selected *GmHATs* and *GmHDACs* in soybean seedlings under five plant hormone treatments. **(A–E)** Relative expression levels of 16 candidate genes (3 *GmHATs* and 13 *GmHDACs*) at different time points following ABA **(A)**, GA **(B)**, IAA **(C)**, MeJA **(D)**, and SA **(E)** treatments. The Y-axis represents the relative fold change in expression levels, normalized using *Actin11* as the reference gene and calibrated against the untreated control group (CK, set to 1). Statistical analyses were performed using *t*-test (* indicates *p* < 0.05, ** indicates *p* < 0.01).

Plant hormones act as signaling molecules, usually at very low concentrations, to regulate plant physiological processes through a complex network of signal transduction (Li et al., 2019a). This delicate coordination mechanism enables plants to flexibly adapt to internal developmental demands and external environmental changes. *Cis-element* analysis of the promoter regions of *GmHAT* and *GmHDAC* revealed the presence of ABA, GA, IAA, MeJA, and SA hormone-responsive elements, suggesting that they may be jointly involved in the regulation of soybean growth and development and stress response processes (**Figure 4**). To further investigate the roles of *GmHAT* and *GmHDAC* in the hormone response mechanism, we treated soybean seedlings with five hormones separately and sampled them at different treatment time periods to detect the expression patterns of the genes in **Figure 6** (**Figure 8**). There were significant differences in the relative expression of all genes at different plant hormone treatment time periods, indicating that all of these genes responded to the five hormone treatments listed above. Although the expression pattern of each gene is not identical, some commonalities and individuality can still be observed. ABA, GA, and IAA treatments resulted in the down-regulation of the expression of most of the genes first and then sustained up-regulation (**Figures 8A–C**). All 16 genes showed significant up-regulation of expression after 24 hours of MeJA treatment (**Figure 8D**). *GmHATs*, RPD3/HDA1, and the two SIR2 subfamily genes showed a consistent trend of up-regulation at all times after SA treatment, but the expression of *GmSRT3* and *GmSRT4* was unchanged at all times (**Figure 8E**). In addition, the expression levels of *GmHAG4* were consistently up-regulated after all four hormone treatments except ABA treatment (**Figure 8**). Notably, all six HD2 subfamily genes showed significant up-/down-regulation of their expression in the five hormone treatments, suggesting that they may be of particular importance in plant hormone response (**Figure 8**). Furthermore, *GmHATs* and *GmHDACs* play important roles in soybean in response to different plant hormones.

## Discussion

Epigenetic modifications mediated by HATs and HDACs are key mechanisms regulating plant development and stress adaptation (Lee and Seo, 2019; Liu et al., 2023; Truong et al., 2021). In this study, we identified 12 *GmHATs* and 28 *GmHDACs* in the soybean genome. Although the number of *GmHats* is comparable to that in *A*.*thaliana* (dicot) and slightly higher than in *O*.*sativa* (monocot), the *GmHDACs* shows significant expansion relative to both species (**Figure 1**). The expansion was most likely driven by two whole-genome duplication (WGD) events in soybean approximately 59 million years ago and 13 million years ago (Schmutz et al., 2010). Phylogenetic and colinearity analyses indicate that segmental duplication played a dominant role in this expansion process, with most duplicate pairs retaining conserved exon-intron structures and functional domains (**Figure 2**). Interestingly, we observed that *GmHDA10* and *GmHDA14* contain significantly fewer introns. As proposed by the “intron delay” hypothesis, genes with fewer introns can be transcribed and processed more rapidly, suggesting that these genes may serve as early responders to environmental stimuli (Heidari et al., 2022).

The diverse expression patterns of *GmHAT* and *GmHDAC* in tissues indicate functional differentiation (**Figure 5**). Consistent with the role of the GCN5-ADA2 complex in root and floral development in *O*.*sativa* and *A*.*thaliana* (Servet et al., 2010; Zhou et al., 2017), our data indicate that *GmHAG1*/*GCN5* is highly expressed in roots, pods, and flowers, suggesting its conserved function in both reproductive and vegetative growth. The enrichment of these development-related *cis-elements* in their promoter regions further supports the notion that these epigenetic modifiers are crucial for the precise temporal regulation of soybean development.

A major finding of this study is the dynamic regulation of histone acetylation under abiotic stress. We identified numerous stress-response *cis-elements* (e.g., MBS, LTR) in the promoters of *GmHAT* and *GmHDAC* (**Figure 4**), which correlated with their transcriptional changes under salt and drought conditions. Notably, *GmHAG4*, *GmHDA16*, and *GmHDT2* were significantly induced throughout the stress treatments (**Figure 6**), mirroring findings in other species (Han et al., 2016; Luo et al., 2012b; Yu et al., 2023).

Previous studies have shown that the plant-specific HD2 family of histone deacetylases primarily targets histone H4 for deacetylation. In *O.sativa*, overexpression of *OsHDT701* resulted in a significant decrease in overall H4K5ac and H4K16ac levels (Ding et al., 2012). *A*.*thaliana* HD2A and HD2B inhibit ABA biosynthesis by deacetylating H4K5ac at the *NCED9* site (Han et al., 2023). The *HD2C* loss-of-function mutant exhibits altered sensitivity to ABA and salt stress (Luo et al., 2012a). *In vitro* enzyme activity assays and mass spectrometry analysis indicate that the HD2 protein possesses broad-spectrum activity in removing acetyl groups from the N-terminal tail of histone H4. In this study, our western blot results revealed that H3K18ac and H4K8ac levels were generally reduced under salt stress and drought stress conditions (**Figure 7**). The deacetylation trend was negatively correlated with the significant upregulation of specific HDAC genes, particularly *GmHDT2*—a member of the HD2 subfamily—whose expression increased more than 60-fold under salt stress conditions. The dramatic upregulation of *GmHDT2* under salt and drought stress correlates with a significant decrease in H4K8ac levels. Given the high conservation of the N-terminal tail structure of H4, we speculate that GmHDT2 may specifically target the H4K8ac site in soybean to regulate stress adaptation. This finding expands our understanding of substrate specificity within the HD2 family.

As endogenous signaling molecules, plant hormones play a crucial role in regulating plant growth and development processes as well as stress responses (Chen et al., 2020b; Wang et al., 2024). Our study demonstrates that *GmHATs* and *GmHDACs* exhibit extensive transcriptional responses to ABA, SA, MeJA, GA, and IAA treatments (**Figure 8**), highlighting the importance of epigenetic regulation in hormonal plasticity. Regarding auxin-mediated development, previous studies have revealed its epigenetic regulatory mechanisms. For instance, HDA9 regulates auxin accumulation during thermomorphogenesis by mediating histone deacetylation at the *YUCCA8* locus (van der Woude et al., 2019). Recently, Pan et al. further demonstrated that HDACs govern shoot organogenesis by regulating H3K9ac and H3K14ac levels in the promoter regions of auxin signaling genes (*ARF19* and *LBD29*) (Pan et al., 2024). Consistent with these findings, the significant induction of specific genes (e.g., *GmHAG4*) under IAA and GA treatments in this study (**Figures 8B, C**) suggests that soybean HATs/HDACs may coordinate hormone-driven developmental processes by dynamically regulating chromatin accessibility.

Under the influence of stress-related hormones, gene expression patterns are particularly noteworthy. Following ABA treatment, numerous genes (e.g., *GmHAG3*, *GmHDT1*) exhibited downregulation at 6 hours, followed by upregulation at 24 hours (**Figure 8A**). This “delayed upregulation” phenomenon suggests the existence of potential feedback regulatory circuits, analogous to the HDA15-MAC3 complex regulating ABA sensitivity via intron splicing (Tu et al., 2022). This hypothesis is further supported by evidence that BdHD1 and AtHD2D regulate ABA responses via histone modifications or protein interactions (Song et al., 2019; Zhang et al., 2022). This aligns with the role of the GCN5-TPL-HDA6 module in jasmonic acid signaling (An et al., 2022) and the involvement of histone deacetylation in ethylene biosynthesis (Deng et al., 2022; Guo, 2022), suggesting that GmHATs and GmHDACs may serve as key regulators in the “growth-defense tradeoff” mechanism. Collectively, these findings demonstrate that the GmHAT and GmHDAC families integrate multi-hormone signaling through dynamic chromatin modifications, forming complex regulatory networks that balance plant survival and development.

This study lays the foundation for deciphering the epigenetic mechanisms underlying soybean stress resistance. Notably, *GmHDT2* was identified as a key candidate gene, whose dynamic changes in H3K18ac and H4K8ac were associated with salt stress and drought responses, offering potential strategies for enhancing stress tolerance. Furthermore, the sensitivity of these genes to multiple hormones indicates their role as signaling hubs integrating environmental and developmental signals, making them potential breeding targets for stress-tolerant crops.

## Conclusions

In this study, we comprehensively identified 12 *GmHATs* and 28 *GmHDACs* genes in soybean, revealing that segmental duplication drove their evolutionary expansion. Expression profiling analysis indicates that these genes are extensively involved in tissue development and hormone signaling pathways. Notably, we confirmed that salt stress and drought stress significantly upregulate specific *GmHDACs* (particularly *GmHDT2*, with a fold-upregulation exceeding 60-fold), while simultaneously reducing genome-wide H3K18ac and H4K8ac modification levels. This study systematically delineates the regulatory landscape of soybean HATs and HDACs, highlighting *GmHDT2* as an ideal candidate gene for developing stress-tolerant crops through epigenetic regulation.

## Data Availability

The original contributions presented in the study are included in the article/**Supplementary Material**, further inquiries can be directed to the corresponding authors.
