# Supplementary material for: Characterization of histone acetyltransferase and histone deacetylase genes under abiotic and hormone stresses in soybean
Source: Front Plant Sci. 2026 Mar 3;17:1753615. doi: 10.3389/fpls.2026.1753615 (PMC12993718; doi:10.3389/fpls.2026.1753615)
Supplement: Supplementary file 1 [file Image1.pdf]

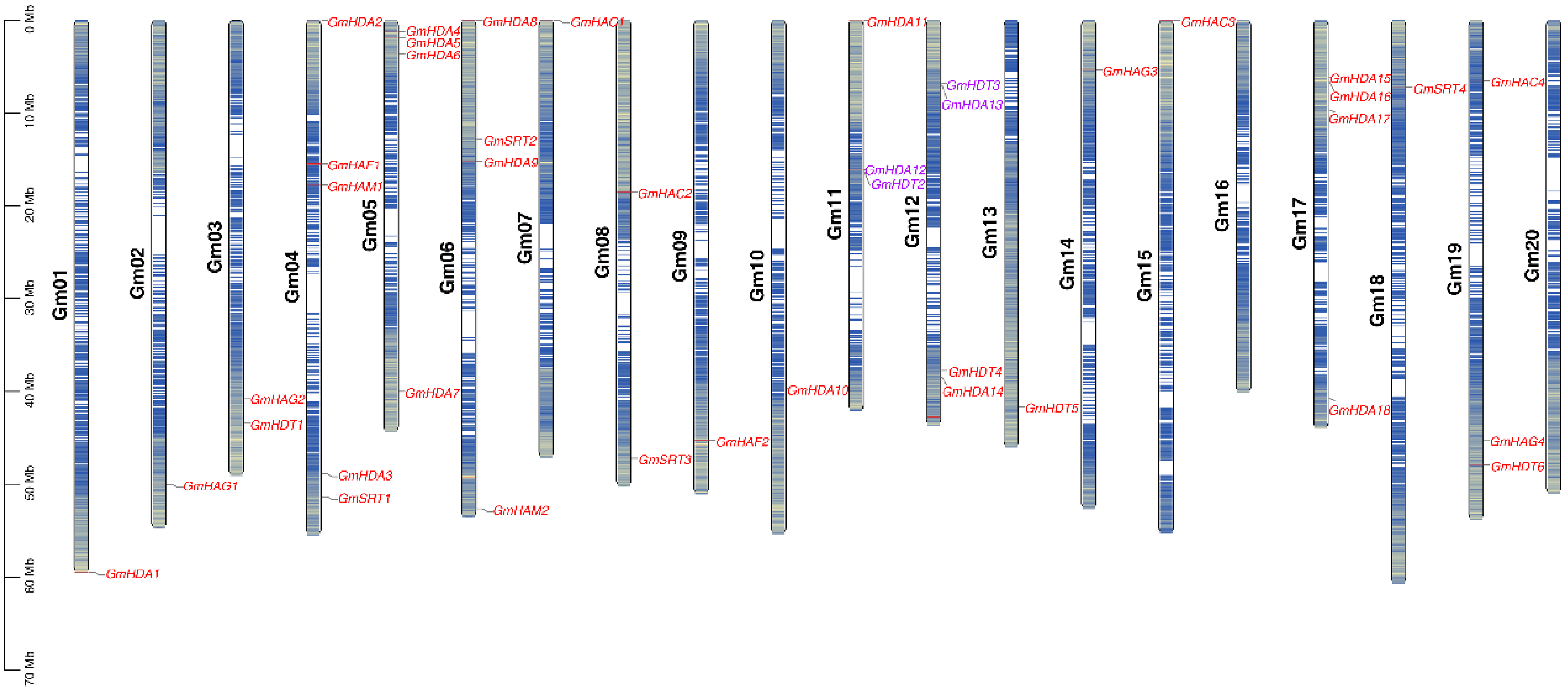

FIGURE S1

Chromosome locations of *GmHAT* and *GmHDAC*. The colored rectangular bars represent the chromosomes of soybean; Gm01-20 represent each corresponding chromosome; purple font represents gene tandem repeat sequences; red denotes gene names, and the 0-700 Mb scale represents chromosome length.
