# Supplementary figures and images for: Characterization of histone acetyltransferase and histone deacetylase genes under abiotic and hormone stresses in soybean
Source: Front Plant Sci. 2026 Mar 3;17:1753615. doi: 10.3389/fpls.2026.1753615 (PMC12993718; doi:10.3389/fpls.2026.1753615)

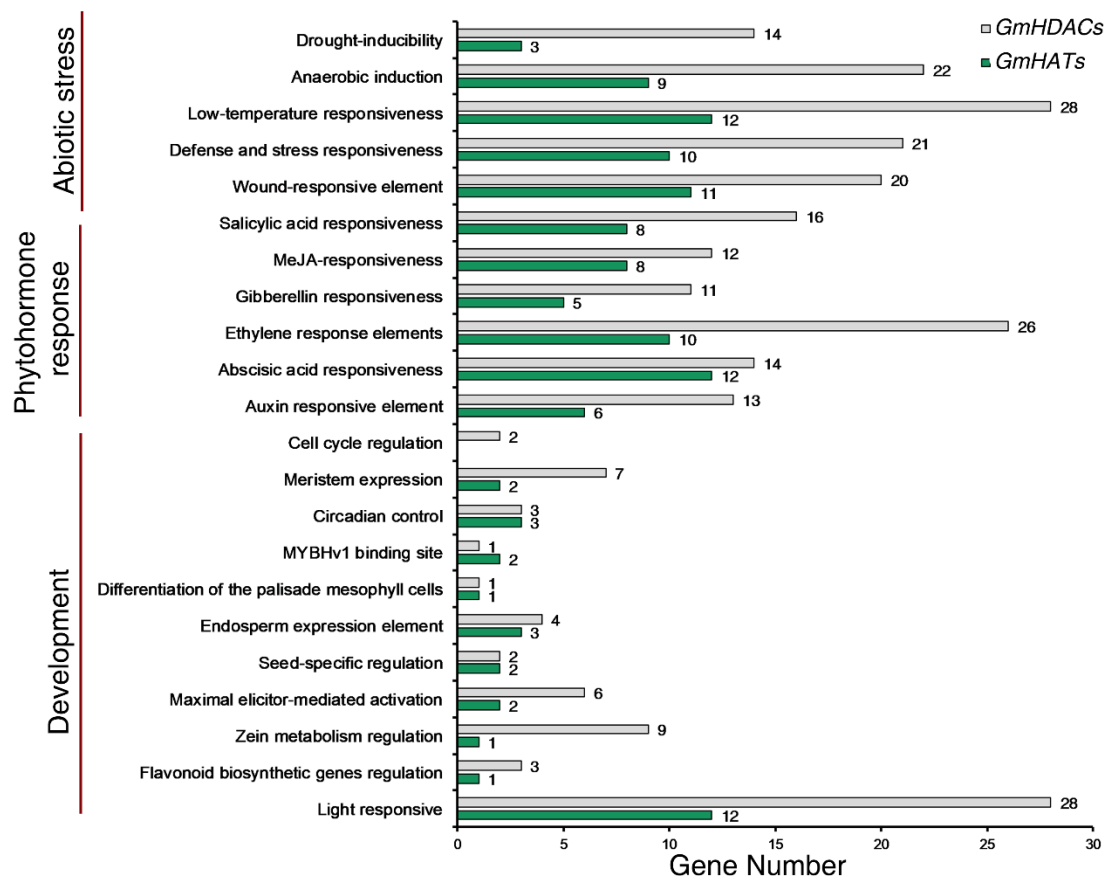

**FIGURE S2**

The number of different types of *cis-elements* contained in *GmHATs* and *GmHDACs*.

Supplement: Supplementary file 2 [file Image2.pdf]
