# Supplementary material for: Characterization of histone acetyltransferase and histone deacetylase genes under abiotic and hormone stresses in soybean
Source: Front Plant Sci. 2026 Mar 3;17:1753615. doi: 10.3389/fpls.2026.1753615 (PMC12993718; doi:10.3389/fpls.2026.1753615)
Supplement: Supplementary file 3 [file Image3.pdf]

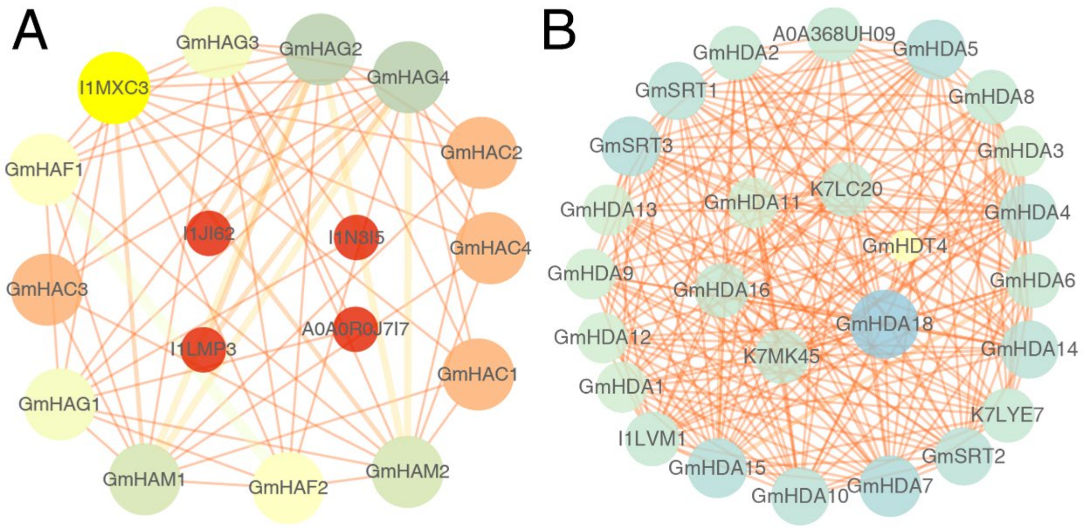

FIGURE S3

GmHATs (**A**) and GmHDACs (**B**) protein interaction networks. The larger the node, the greater the number of proteins that may interact. Thicker line segments indicate higher feasibility of interactions.
